# GALLIUM trial: the tortoise (rituximab) and the hare (obinutuzumab) race

**DOI:** 10.4155/fsoa-2018-0122

**Published:** 2019-02-12

**Authors:** Ricardo García-Muñoz, Judit Anton-Remirez, María Josefa Nájera, Elena Gutierrez-Gamarra, Raisa Peralta, Jesús Feliu

**Affiliations:** 1 Department of Hematology, Hospital San Pedro, Logroño, La Rioja, Spain; 2 Department of Physical Medicine & Rehabilitation, Complejo Hospitalario de Navarra, Pamplona, Spain; 3 Department of Hematology, Hospital Poniente, Almería, El Ejido, Andalucía, Spain

**Keywords:** ADCC, follicular lymphoma, NK-cell, obinutuzumab

## Is the lack of effector natural killer (NK) cells responsible for the discrete benefits of obinutuzumab compared with rituximab in clinical trials?

Obinutuzumab is a glycoengineered, Type II anti-CD20 molecule with less complement-dependent cytotoxicity than Type I anti-CD20 rituximab but with greater antibody-dependent cellular cytotoxicity (ADCC) [1]. Although obinutuzumab seems to be more potent than rituximab *in vitro* [2], the randomized Phase II GAUSS study that compared single-agent obinutuzumab with rituximab has not generated encouraging early results [3]. In the Phase III GALLIUM trial (clinicaltrials.gov identifier NCT0132968), obinutuzumab plus chemotherapy significantly prolonged progression-free survival in previously untreated patients with follicular lymphoma compared with rituximab plus chemotherapy after a median follow-up of 3 years [4–6]. Yet, overall survival was equivalent between the two arms [4–6]. That trial randomly assigned patients treated with standard induction chemotherapy to either rituximab or obinutuzumab [4,5]. Patients who responded to treatment continued with 2 years of maintenance with the same antibody assigned at induction [4,5]. In this perspective, we describe the immunological aspects that affect the conclusions of clinical trials that compare the two anti-CD20 monoclonal antibodies.

## Should obinutuzumab replace rituximab as the preferred antibody for initial treatment of follicular lymphoma?

The first observation to help answer this question is that complete response (CR) rates at the end of induction showed no significant differences between obinutuzumab and rituximab for any of the chemotherapy combinations with cyclophosphamide, doxorrubin, vincristine and prednisone (CHOP), bendamustine (B) or cyclophosphamide, vincristine and prednisone (CVP) [5]. Moreover, administration costs of obinutuzumab are substantially higher when compared with rituximab [6]. Although these observations are sufficient to conclude that rituximab remains the preferred antibody in the induction treatment of patients with *de novo* follicular lymphoma [6], indirect immunological observations may indicate the contrary [7,8].

Interestingly, CR rates in the BRIGHT study [9] can be compared with the Phase III trial of BR versus R-CHOP in patients with indolent non-Hodgkin lymphomas (NHL) and mantle cell lymphoma (MCL) performed by the Study Group for Indolent Lymphomas (StiL) trial in Germany [10]. In both studies, the CR rate of BR was superior to that of R-CHOP (Study Group for Indolent Lymphomas BR 40% vs R-CHOP 30% and in BRIGHT BR 40% vs R-CHOP 26%). The GALLIUM study showed no significant differences in CR rate in R-chemotherapy combinations (BR 61% vs R-CHOP 61%), in O-chemotherapy combinations (OB 63% vs O-CHOP 66%) or when comparing O-chemotherapy versus R-chemotherapy [5].

## If we assume that bendamustine and CHOP are similar in CR rate, the question that arises is why can obinutuzumab not increase the CR rate in clinical trials if *in vitro* it is superior when compared with rituximab?

The first clue is that obinutuzumab needs a fit immune system to cause an effect; it especially depends on NK cells to induce an ADCC effect, but we and others have demonstrated that obinutuzumab itself induces depletion of NK cells after the first-dose infusion [7,8]. This fact evidently benefits the rituximab arm, because rituximab uses complement to act and has only a modest ADCC effect. This also may be the explanation for the results in the Phase II GAUSS study [3].

The second clue is that, surprisingly, several patients show low NK cell counts in peripheral blood and this ‘immunologic pretreatment signature’ is associated with inferior overall survival in patients with follicular lymphoma [11]. Again, this patient characteristic may hinder the action of obinutuzumab and benefit the rituximab arm, because complement-dependent cytotoxicity is not largely impaired in follicular lymphoma patients. Unfortunately, no information about NK cells or complement during the GALLIUM trial has been given [5].

The third clue is that chemotherapy, especially bendamustine, induces an important reduction of NK cells, again favoring the rituximab arm [12]. Despite this initial destruction of NK cells in the first cycle of obinutuzumab–bendamustine, the combination may be of benefit to the patient by the initial first cycle NK-ADCC effect. Unfortunately, bendamustine also may be associated with increased risk of infections and severe adverse events [5,6]. We speculate that this NK cell destruction may be another explanation for the similar results in the CR rate of rituximab–bendamustine and obinutuzumab–bendamustine, because without this first obinutuzumab-NK-ADCC cycle, obinutuzumab plus bendamustine may be similar to a cycle of bendamustine alone when NK cells are too low to be efficient. Importantly, the benefit of bendamustine is a double-edged sword. Bendamustine may destroy CD4^+^ helper T cells that may support follicular lymphoma; however, it may diminish CD4^+^ T cells that may protect against infections and second neoplasms [5,6,12].

The fourth clue is that similar results in the CHOP arms indicate that after a first obinutuzumab-NK-ADCC effect, the next cycles lost the power of the obinutuzumab-induced ADCC effect [5,6]. However, the less potent action of rituximab is permanent in all the cycles, as we observe a similar balance after the end of induction treatment [5,6].

In summary, the low pretreatment NK cells seen in some patients with high-risk follicular lymphoma [2,11], the mechanism of action of obinutuzumab itself [1,2,7,8] and the influence of chemotherapy partners [5,6,8,12] benefit rituximab outcomes to the detriment of obinutuzumab.

At this point, induction immune-chemotherapy would be expected to obtain similar results with either obinutuzumab or rituximab combined with chemotherapy because rituximab arms are privileged. However, the conclusion of the GALLIUM study is that improved progression-free survival was observed for obinutuzumab plus chemotherapy [4–6]. Importantly, the most compelling rationale to recommend obinutuzumab over rituximab would be a 34% reduced risk of a progression event at 2 years [4–6]. We speculate that during maintenance treatment, some patients may recover their NK cells [8,12] and maintain better immune surveillance to destroy the residual tumoral cells that cannot be killed by immunochemotherapy induction more efficiently than rituximab [2]. Alas, this variable was not controlled during the GALLIUM study [5] and, in general, the perception is that the advantage of obinutuzumab over rituximab is small [6]. However, patient-derived expanded NK cells armed with obinutuzumab may be a reasonable therapeutic strategy and should be explored in a similar way to other clinical trials with lymphokine-activated cells (LAK cells) and rituximab (clinicaltrials.gov identifier NCT01329354) [13].

In conclusion, combination of obinutuzumab with NK cells may help optimize the potential of this novel anti-CD20 monoclonal antibody.
